# Evaluation of an intervention to improve waterless surgical hand antisepsis accuracy in a tertiary hospital of East China: a prospective pre-post intervention study

**DOI:** 10.3389/fpubh.2025.1583510

**Published:** 2025-09-11

**Authors:** Yonghui Ma, Xia Yue, Ning Li, Jixia Wang, Juan Wang, Yujie Xia, Xing Zhang, Min Zhang, Peng Wang, Shuangshuang Wang, Luhan Wen, Zhenghui Liu, Dingding Zhang, Tong Zhang, Lili Liu, Li Kong, Yusen Li, Yue Zhang, Yirong Wang, Xiujuan Meng

**Affiliations:** ^1^Hospital Infection Management Department, Affiliated Hospital of Jining Medical University, Jining, China; ^2^Jining Medical University, Jining, Shandong, China

**Keywords:** waterless, surgical hand antisepsis, tertiary hospital, China, surgical site infections (SSIs)

## Abstract

**Background:**

Surgical site infections (SSIs) are the most frequent type of healthcare-associated infections (HAIs) in low- and middle-income countries (LMICs). Effective surgical hand antisepsis is a critical step in the prevention of SSIs. Limited research on clinician interventions regarding accuracy in waterless surgical hand antisepsis (WSHA) is available. This study evaluated the effect of a tailored, multifaceted improvement strategy on WSHA in a tertiary hospital in East China. We also performed a process evaluation to explore the mechanisms through which our strategy brought about change.

**Methods:**

A prospective, pre-post intervention study was performed from January 2024 through December in 28 departments. Data from the pre-post intervention studies were collected using a specially designed score checklist and video surveillance (≥4 per department). In addition to the score collection, the mid-term assessment also used a questionnaire that included demographic characteristics and cultural climate surveys. A number of customized interventions were conducted before and after the mid-term assessment.

**Results:**

The scores of pre-intervention, mid-term assessment, and post-intervention were 71.4 ± 16.8, 92.7 ± 9.2, and 78.7 ± 19.4, respectively. There were statistical differences between the scores of pre-intervention and mid-term assessment (*p* < 0.001), mid-term assessment and post-intervention (p < 0.001), and pre-intervention and post-intervention (*p* = 0.002). There were statistical differences among different genders, ages, positions, years of working experience, and departments (*p* < 0.05). Lower scores appeared in males, orthopedics, and internal medicine. The psychological comfort score was more than 4 points when being reminded to standard WSHA. The number of times the score was less than 2 points was reduced.

**Conclusion:**

Our interventions have been successful in improving WSHA accuracy. In the future, it will be necessary to closely monitor and supervise WSHA practices to determine the long-term effectiveness of current intervention strategies.

## Introduction

Surgical site infections (SSIs) are the most frequent type of healthcare-associated infections (HAIs) in low- and middle-income countries (LMICs) (1), and the second most common type of HAI in Europe and the US (2). SSIs can have a substantial economic cost for both the patient and health services, leading to rising mortality and healthcare costs (3). In 2017, a French cohort highlighted the mean cost of each SSI treatment to be around €1,814; the same year, the Centers for Disease Control and Prevention guidelines evaluated the mean cost of SSI treatment to range from $10,443 to $25,546 per SSI (4). The prevention and control of SSIs is crucial.

Various interventions have been used to prevent SSIs, such as prophylactic use of antibiotics (5), preoperative skin preparation (6), glycemic control (7), maintaining normal body temperature (8), etc. Hand hygiene is the single most effective strategy for reducing HAIs and the transmission of antimicrobial drug-resistant pathogens. Similarly, effective surgical hand antisepsis is a critical step in the prevention of SSIs in surgical patients. Waterless hand scrub is as effective as traditional hand scrub in cleansing the hands of microorganisms and more efficient in terms of scrub time (9). At the same time, it can save millions of liters of water annually, thereby reducing the hand surgeon’s contributions to resource overuse and water scarcity (10).

The WHO Guidelines on ‘Hand Hygiene in Healthcare Settings’ (11) and ‘Specification of Hand Hygiene for Healthcare Workers (WS/T 313–2019)’ (12) refined the surgical hand antisepsis methods. In the context of this paper, ‘waterless surgical hand antisepsis (WSHA)’ means that before the operation, medical staff rub and rinse their hands, forearms, and the lower 1/3 of the upper arm with running water and hand sanitizer, then use a waterless antiseptic agent to remove or kill temporary bacteria and reduce resident bacteria on the hands and forearms, up to the lower 1/3 of the upper arm. Compliance with the correct technique for all these elements has been challenging, despite the adoption of surgical hand antisepsis policies by most institutions. Limited data are available on clinicians’ adherence to WSHA guidelines.

The aim of this study was to investigate the current situation of WSHA, analyze the influencing factors, formulate feasible intervention measures, and then evaluate the effect of these intervention measures. It is assumed that efforts to improve professional practice are more likely to succeed when the individual and contextual factors that shape current practice and hinder or enable improvement are identified and considered (13). We also performed a mid-term assessment and questionnaire survey to measure participants’ exposure to the intervention measures and adjust new interventions in a timely manner.

## Materials and methods

### Study design and setting

This prospective, pre-post intervention study was conducted from January 2024 through December 2024 in a tertiary hospital in East China. This hospital is a third-grade class A general hospital that integrates medical treatment, teaching, scientific research, preventive healthcare, and guidance at the grassroots level. Currently, the hospital comprises two hospital areas with a total of 4,100 open beds, encompassing 24 surgical departments. It is responsible for medical and healthcare services for over 20 million individuals. Annually, the hospital accommodates 4 million outpatient visits and 200,000 inpatients, with an average length of stay of 6.4 days. The hospital infection management department has 18 full-time infection control staff, all of whom have passed the provincial infection control examination and obtained the post qualification. Responsibilities are clearly defined with an equal division of labor among internal medicine, surgery, the intensive care unit, and the outpatient department.

In addition to routine surgeons, the study population included clinicians who perform invasive procedures in internal medicine, such as cardiology, neurology, and nephrology, as some of their procedures also require surgical hand antisepsis.

**Figure 1:**
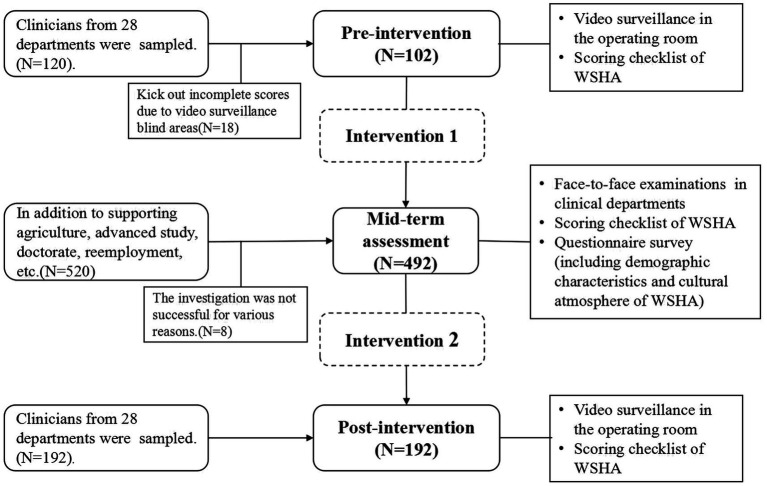
The study flow chart.

This study was conducted in three phases: pre-intervention (January–February), mid-term assessment (June–July), and post-intervention (November–December) (Figure 1).

### Data collection tools

According to the ‘Specification of Hand Hygiene for Healthcare Workers (WS/T 313-2019),’ we formulated the scoring checklist for the WSHA operation process on a scale of 100. The checklist consists of two parts: hand washing and hand antisepsis. The hand-washing section consists of 8 steps with 13 scoring items. The hand disinfection section consists of 12 steps with 16 scoring items. The score was divided into ‘yes’ and ‘no’, the operation is scored correctly if answered ‘yes,’ and vice versa.

A questionnaire was also used to gather demographic characteristics and assess the cultural atmosphere of WSHA. After consulting relevant literature (14), the cultural atmosphere questionnaire was designed, including 4 questions. The psychological feeling of reminding or being reminded ranged from ‘very uncomfortable’ to ‘very comfortable’ on a scale of 1–5. The number of times reminding or being reminded in the past 2 months ranged from ‘0–5 times’ to ‘more than 16 times’ on a scale of 1–5. A psychological comfort score of more than 3 points and the number of scores greater than 2 points were defined as indicating a good cultural atmosphere (15).

### Data collection process

The 28 departments selected were randomly assigned to 15 professionally trained and qualified full-time infection control staff. Before and after the intervention, the full-time infection control staff randomly checked the surveillance video in the operating room according to the randomly assigned departments. No less than 4 people were randomly selected from each department. The scores were recorded according to the scoring checklist. The observed staff were not given prior notice; Meanwhile, the spot-check personnel could not recognize them because the observed personnel were wearing hand-washing clothes, hats, and masks.

The mid-term assessment was conducted in the form of a face-to-face evaluation by full-time infection control staff in clinical departments, and all clinicians who performed WSHA were assessed. In addition to collecting scores from on-site assessments, questionnaires were also conducted among the participants.

### Intervention

Interventions 1 were developed based on pre-intervention baseline surveys (March to May):

- Hardware facility: Install and adjust video surveillance to cover all blind areas.
- Change dry hand position: Move from the operating room to the sink.
- Change the dry hand method: Switch from sterile towels to paper towels.
- Training and education: Create training videos on WSHA operation methods, and post them on hospital websites and mobile public platforms for training and educational publicity.
- 5 May – World Hand Hygiene Day: Publicity activities-Preach the importance of WSHA and the harm of SSI.

Interventions 2 were developed based on mid-term assessments and questionnaires (August to October):

- Hardware facility: A stopwatch timing device was installed near the pool.
- Supervision system: The supervision plan for full-time staff and the self-examination plan for operating room staff were formulated.
- Special training: Targeted training for different groups, such as male clinicians, orthopedic departments, internal medicine departments, etc.
- Reward and punishment system: Incentive and punishment mechanisms were developed, linked to staff performance.
- 15 October – Global Handwashing Day: Organizing a WSHA skills competition.

### Ethical considerations

This study was approved by the Ethics Committee of the Hospital. Written informed consent was obtained from all participants before they completed the questionnaire. Additionally, participants were informed about the voluntary nature of their participation, the confidential handling of their data, and the purpose of the study.

### Data analysis

Statistical analysis was conducted using the Statistical Package for the Social Sciences (version 25). Descriptive statistics were used to summarize all parameters, including means, standard deviations, frequencies, and percentages. Additionally, the Chi-square test was used to compare the rates between different groups, and statistical significance was assumed at *p* < 0.05.

## Results

### Pre-intervention

A total of 120 clinicians were randomly sampled for the baseline survey before the intervention. Eighteen were excluded due to the blind area of the video surveillance and incomplete score lists, leaving 102 for analysis. The score for WSHA was 71.4 ± 16.8 (Table 1). The steps with low accuracy were reflected in the following: the method of rubbing finger seams (66.7%), thumbs (58.8%), fingertips (58.8%), and keeping hands higher than elbows (64.7%) in the hand-washing section. Meanwhile, fingertip immersion (right: 31.4%, left: 31.4%), arm rubbing time (right: 68.6%, left: 66.7%), and circular motion around the hands (right: 29.4%, left: 33.3%) were problematic in the hand antisepsis section (Table 2).

**Table 1:** Implementation of WSHA before and after intervention.

Stages	Score (mean±SD)	P
Pre-intervention (*n* = 102)	71.4 ± 16.8	<0.001^a^
Mid-term assessment (*n* = 492)	92.7 ± 9.2	<0.001^b^
Post-intervention (*n* = 192)	78.7 ± 19.4	0.002^c^

a: Comparison between pre-intervention and mid-term assessment. b: Comparison between mid-term assessment and post-intervention. c: Comparison between pre-intervention and post-intervention.

**Table 2:** The scoring checklist and implementation of the WSHA.

Scoring checklist		Pre-intervention (*n* = 102)	Mid-term assessment (*n* = 492)	Post-intervention (*n* = 192)
		*n* (%)	*n* (%)	*n* (%)
Hand Washing	1. Rinse hands and forearm up to the lower third of the upper arm under running water. (3 points)	100 (98.0)	483 (98.2)	190 (99.0)
	2. Apply an appropriate amount of hand sanitizer to cover the entire palm, back of the hand, fingers, and interphalangeal area. (3 points)	88 (86.3)	428 (87.0)	176 (91.7)
	3. Use a nail cleaner to remove the dirt from under the nails and a brush to clean the wrinkles of the hand skin. (3 points)	102 (100.0)	492 (100.0)	192 (100.0)
	4. Hand washing and rubbing method:			
	4.1 Place the palms facing each other with fingers together, then rub them together. (3 points)	90 (88.2)	465 (94.5)	178 (92.7)
	4.2 Rub the palms together along the backs of the fingers, then exchange hands. (3 points)	84 (82.4)	461 (93.7)	166 (86.5)
	4.3 Place the palms facing each other and interlace the fingers, rubbing them together. (3 points)	68 (66.7)	441 (89.6)	162 (84.4)
	4.4 Bend the fingers so the joints rotate and knead in the other palm, then exchange hands. (3 points)	74 (72.5)	447 (90.9)	170 (88.5)
	4.5 Use the right hand to rotate and rub the left thumb, then exchange hands. (3 points)	60 (58.8)	473 (96.1)	150 (78.1)
	4.6 Place the tips of the five fingers in the palm of the other hand, rotate, rub, and exchange hands. (3 points)	60 (58.8)	483 (98.2)	144 (75.0)
	5. Rotate and rub the forearm to the lower third of the upper arm. (3 points)	74 (72.5)	467 (94.9)	160 (83.3)
	6. Rinse the hands, forearms, and lower third of the upper arms under running water. (3 points)	102 (100.0)	482 (98.0)	188 (97.9)
	7. Keep the hands on the chest, above the elbows. (5 points)	66 (64.7)	486 (98.8)	176 (91.7)
	8. Use hand drying supplies to dry the hands, forearms, and lower third of the upper arms. (3 points)	98 (96.1)	467 (94.9)	182 (94.8)
Hand Antisepsis	1. Take an appropriate amount of hand disinfectant and place it in the palm of the left hand. (3 points)	98 (96.1)	478 (97.2)	184 (95.8)
	2. Immersed the right fingertip in hand disinfectant for ≥5 s. (5 points)	32 (31.4)	393 (79.9)	88 (45.8)
	3. Apply the disinfectant to the right hand, forearm and lower third of the upper arm.(3 points)	96 (94.1)	461 (93.7)	176 (91.7)
	4. Use a circular motion to rub the disinfectant around the forearm to the lower third of the upper arm.(4 points)	30 (29.4)	447 (90.9)	96 (50.0)
	5. Completely cover the skin area with the disinfectant, and continue rubbing for 10–15 s until the disinfectant is dry. (5 points)	70 (68.6)	404 (82.1)	146 (76.0)
	6. Take an appropriate amount of hand disinfectant and place it in the palm of the right hand. (3 points)	100 (98.0)	480 (97.6)	172 (89.6)
	7. Repeat the disinfection process for the right hand, and immersing the tip of the left finger in the disinfectant for ≥5 s. (5 points)	32 (31.4)	421 (85.6)	86 (44.8)
	8. Apply the disinfectant to the left hand, forearm, and lower third of the upper arm. (3 points)	98 (96.1)	471 (95.7)	176 (91.7)
	9. Use a circular motion to rub the disinfectant around the forearm up to the lower third of the upper arm. (4 points)	34 (33.3)	461 (93.7)	106 (55.2)
	10. Completely cover the skin area with the disinfectant, and continue rubbing for 10–15 s until the disinfectant is dry. (5 points)	68 (66.7)	414 (84.1)	130 (67.7)
	11. Take an appropriate amount of hand disinfectant, place it on the palms, and rub the hands up to the wrists. (4 points)	86 (84.3)	479 (97.4)	160 (83.3)
	12. Method of rubbing:	94 (92.2)	473 (96.1)	166 (86.5)
	12.1 Place the palms facing each other with the fingers together, then rub them together. (3 points)	78 (76.5)	470 (95.5)	142 (74.0)
	12.2 The palms of the hands are rubbed together along the backs of fingers, then exchanged. (3 points)	70 (68.6)	471 (95.7)	146 (76.0)
	12.3 Place your palms facing each other and interlace your fingers, then rub them together. (3 points)	80 (78.4)	463 (94.1)	156 (81.3)
	12.4 Bend the fingers so that the joints rotate and knead the other palm, then exchange hands. (3 points)	68 (66.7)	472 (95.9)	134 (69.8)
	12.5 Use the right hand to rotate and rub the left thumb, then exchange hands. (3 points)	100 (98.0)	483 (98.2)	190 (99.0)

n(%): Frequency and constituent ratio of correct execution.

### Mid-term assessment and investigation

A total of 492 clinicians were examined and surveyed on the spot. The score for WSHA was 92.7 ± 9.2. There was a statistically significant difference (*p* < 0.001) compared to before the intervention (Table 1). The low accuracy rates in the mid-term assessment were mainly reflected in steps with time requirements, such as soaking fingertips and rubbing arms (Table 2).

Among the surveyed clinicians, more than half were male (68.5%). Those aged ≤35 years comprised the largest age group (42.7%). Most clinicians (81.5%) held a master’s degree. Attending physicians accounted for 42.1%. Over half (52.4%) of the clinicians had worked for more than 10 years. A minority of clinicians were from internal medicine departments (6.7%) (Table 3).

**Table 3:** Comparison of the demographic characteristics and implementation of the mid-term assessment (*n* = 492).

Items		*n* (%)	Score (mean ± SD)	p
Gender	Male	337 (68.5)	91.47 ± 9.67	<0.001
	Female	155 (31.5)	95.46 ± 7.49	
Age (years)	≤35	210 (42.7)	93.4 ± 9.2	0.023
	36–45	206 (41.9)	93.1 ± 8.9	
	46–55	68 (13.8)	90.2 ± 10.1	
	≥56	8 (1.6)	87.4 ± 7.6	
Educational level	Bachelor or below	50 (10.2)	93.20 ± 7.99	0.786
	Master	401 (81.5)	92.76 ± 9.29	
	Doctor	41 (8.3)	91.88 ± 10.11	
Position	Resident physician or below	109 (22.2)	92.10 ± 10.07	0.036
	Attending physician	207 (42.1)	93.97 ± 8.36	
	Associate chief physician	122(24.8)	92.30 ± 9.41	
	Archiater	54 (11.0)	90.22 ± 9.70	
Years of working experience	0–3 years	106 (21.5)	92.12 ± 10.10	0.046
	3–5 years	33 (6.7)	96.97 ± 4.85	
	5–10 years	95 (19.3)	93.63 ± 8.72	
	10–15 years	122 (24.8)	92.28 ± 9.84	
	15 or more years	136 (27.6)	91.94 ± 8.87	
Years of participation in surgery (surgical hand antisepsis)	0–3 years	92 (18.7)	92.38 ± 9.81	0.276
	3–5 years	61 (12.4)	94.51 ± 7.63	
	5–10 years	112 (22.8)	93.62 ± 9.22	
	10–15 years	102 (20.7)	92.13 ± 10.01	
	15 or more years	125 (25.4)	91.81 ± 8.77	
Department	Obstetrics and Gynecology (including Reproductive Medicine)	110 (22.4)	96.52 ± 5.63	<0.001
	Department of orthopedics	66 (13.4)	84.98 ± 11.88	
	Department of Five Senses	68 (13.8)	93.76 ± 8.91	
	Department of general surgery	89 (18.1)	95.60 ± 5.13	
	Internal medicine	33 (6.7)	87.09 ± 13.73	
	Neurosurgery	27 (5.5)	90.26 ± 9.50	
	Cardiothoracic surgery department	38 (7.7)	92.26 ± 7.39	
	Urology	20 (4.1)	94.30 ± 5.59	
	Others	41 (8.3)	92.90 ± 8.52	

Department of Five Senses: Otolaryngology, oral and maxillofacial surgery, ophthalmology, etc.; Department of general surgery: thyroid surgery, breast surgery, hepatobiliary surgery, gastrointestinal surgery, vascular surgery, anorectal surgery, etc.; Internal medicine: Neurology, cardiology, nephrology, pain, etc.; Others: pediatric surgery, burn plastic surgery, emergency surgery, emergency stroke, etc.

The study compared WSHA scores among clinicians with different demographic characteristics. There were statistical differences based on gender, age, position, years of working experience, and department. Higher scores were found in females, those aged ≤35 years, attending physicians, those with 3–5 years of work experience, and clinicians in obstetrics and gynecology (including reproductive medicine) and general surgery departments. Lower scores were observed in the orthopedics and internal medicine departments (Table 3).

**Figure 2:**
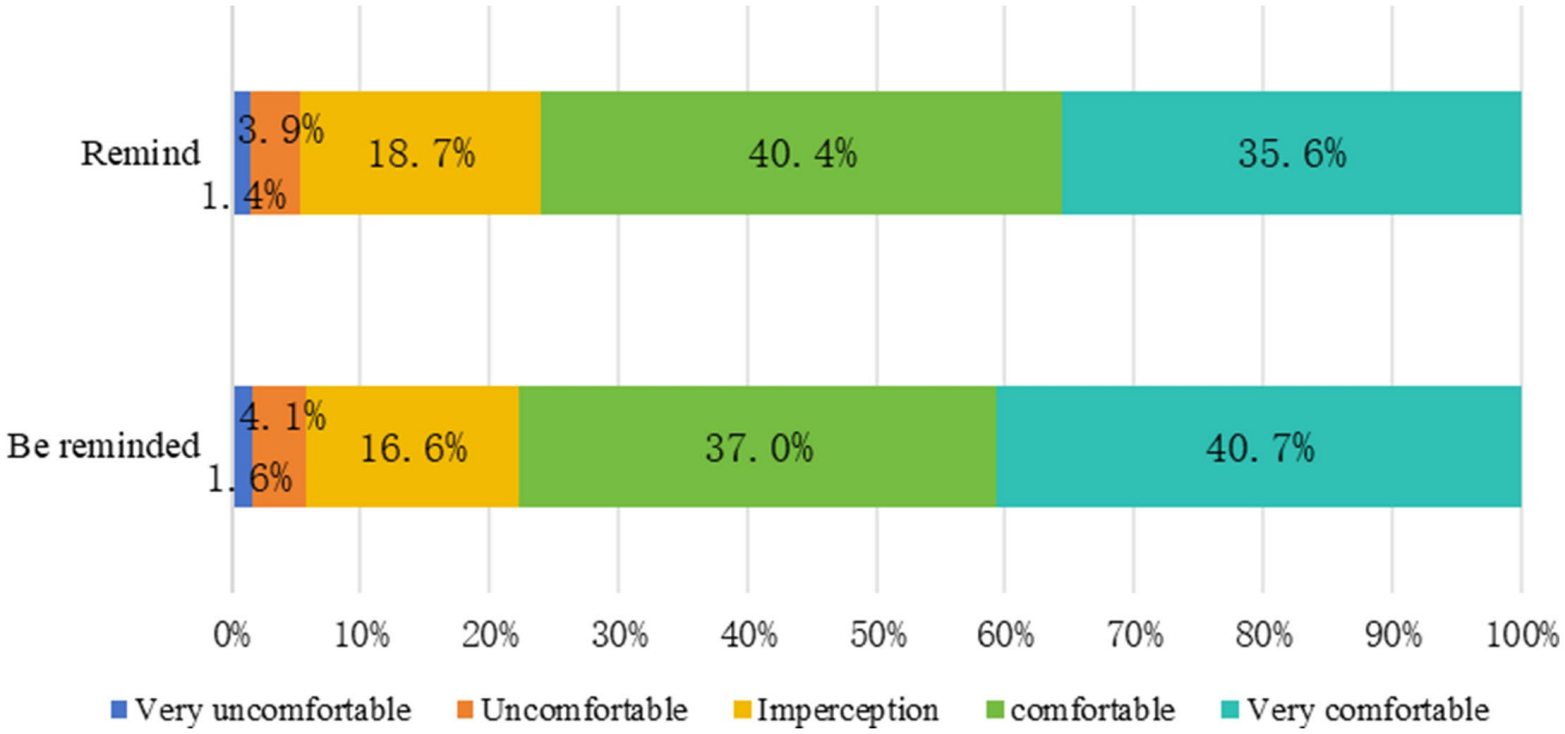
The constituent ratio of psychological feelings between the person reminding and the person being reminded in standardizing the implementation of WSHA.

**Figure 3:**
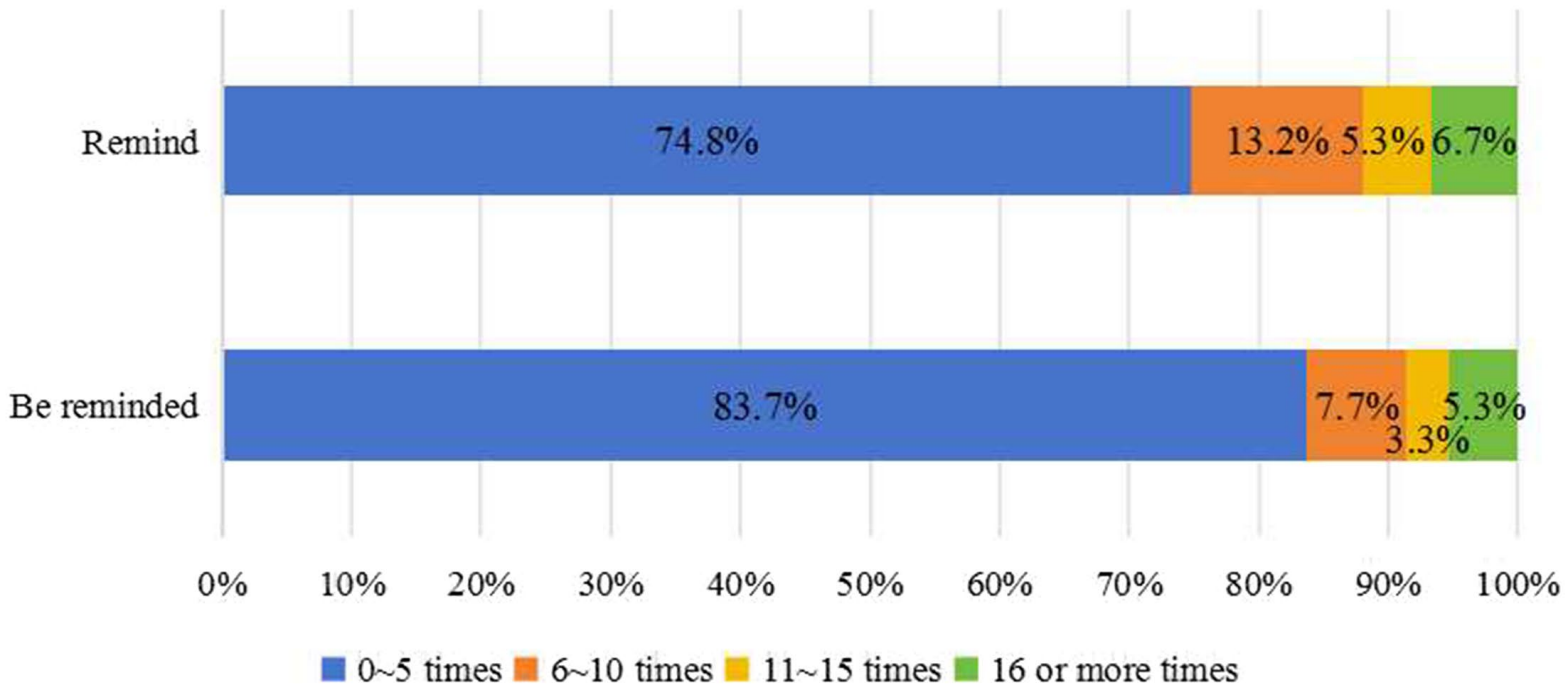
The constituent ratio of the frequency of reminding and being reminded in standardizing the implementation of WSHA.

Figures 2, 3 show the constituent ratios of psychological feelings and the number of times of reminding and being reminded to standardize the implementation of WSHA, respectively. The psychological feelings of reminding and being reminded as comfortable or above accounted for 76.0 and 77.7%, respectively. In the past 2 months, reminding and being reminded more than six times accounted for 25.2 and 16.3%, respectively.

Table 4 shows the scores from the WSHA cultural atmosphere survey. The psychological comfort score was more than 4 points when being reminded or reminded to follow the WSHA standard. The number of times score was less than 2 points.

**Table 4:** Investigation of the cultural atmosphere of WSHA (*n* = 492).

Items	Score (mean±SD)
The psychological experience of being reminded to standardize the implementation of WSHA.	4.1 ± 0.9
The psychological experience of reminding others to standardize the implementation of WSHA.	4.1 ± 0.9
The number of times I have been reminded to standardize the number of WSHA in the past 2 months.	1.3 ± 0.8
The number of times I have reminded others to standardize the number of WSHA in the past 2 months.	1.4 ± 0.9

### Post-intervention

After the intervention, 192 people were randomly examined. The score for WSHA was 78.7 ± 19.4 (Table 1). There were statistically significant differences (*p* < 0.05) in WSHA scores between the mid-term examination and post-intervention, as well as between pre-intervention and post-intervention (Table 2).

## Discussion

Surgical hand antisepsis can eliminate transient flora and reduce resident hand flora. The effective implementation of this simple and inexpensive method is crucial to preventing the transfer of HAIs (16, 17). In this study, a pre-post intervention was carried out to assess WSHA in a tertiary hospital in LMICs.

The advantages of this study included the use of a double-blind method before and after the intervention. The observers were unaware of the spot-check timing, and the clinicians did not know the observer. In addition, mid-term assessment and questionnaires allowed for adjustments to interventions.

This study found that the baseline survey scores before the intervention were lower (71.4 ± 16.8), which was consistent with a study conducted in an operating room on surgeon behavior and knowledge of hand scrub (18). Two major problems were identified in the survey, the first being the rubbing method. We developed a training video to promote the hand rubbing technique, allowing for cyclic viewing and learning compared to on-site training. The second issue was that hands were not kept higher than the elbows after washing, which was consistent with previous research (19).

Before the intervention, clinicians typically performed dry hand disinfection by entering the operating room after washing their hands and waiting for the instrument nurse to provide sterile towels. This process not only resulted in wet floors in the operating room but also caused delays in obtaining sterile towels. Additionally, some clinicians retrieved sterile towels from their own aseptic instrument kits, which risked contaminating the kits and allowed water to flow from the elbow to the hand. Some studies have found no difference in the effectiveness of sterile towels and toilet paper in preventing WSHA and suggest that using toilet paper can reduce hospital operating costs (20). Huang et al. (21) compared different hand-drying materials and found that toilet paper could dry hands quickly, has good water absorption, and helps maintain the concentration of hands-free disinfectants, improving their adhesion to the skin. To address these issues, we improved the hand-drying process by replacing sterile towels with toilet paper and relocating the drying station from the operating room to the sink. This ensures that clinicians can dry their hands immediately after washing, without waiting.

The WSHA score in the mid-term assessment was higher than that before the intervention (*p* < 0.05). In addition to the effectiveness of the intervention, the Hawthorne effect may have influenced the results. Due to on-site observation, clinicians may have changed their behavior, masking the true performance of WSHA (22) and introducing bias (23) that video surveillance may help avoid (24). Additionally, on-site assessments were conducted during free time, allowing ample time to complete WSHA properly.

Analysis of different factors, such as gender, age, position, years of work experience, and department, revealed variations in assessment scores. Several studies have shown gender differences in hand hygiene compliance among healthcare workers, with men generally having lower compliance rates than women (25, 26). This study found a similar pattern in WSHA, which may be related to inherent gender differences, as female clinicians tend to be more careful and patient. Further research is needed on the psychosocial determinants of WSHA, similar to studies on hand hygiene (27).

Orthopedic departments received the lowest scores, followed by internal medicine departments that require WSHA. Most orthopedic clinicians are male, and the high surgical workload may contribute to lower compliance, as surgeons may rush through procedures. The SSI rate in orthopedics remains a major problem, especially since infections involving implanted biomaterials are particularly difficult to treat. The prevention of SSI in orthopedics is a critical challenge (28), and careful implementation of preventive measures, including WSHA, is essential to reducing its incidence. In recent years, interventional procedures such as cardiac interventions (29) have become increasingly common, highlighting the need for rigorous infection prevention and control measures to avoid adverse events. In response, we conducted targeted training for low-scoring departments, such as orthopedics, emphasizing the risks of SSIs and the importance of proper procedures of WSHA.

In addition to the influence of demographic characteristics, steps with time requirements were performed less well. To address this, we introduced a stopwatch device to improve timing accuracy. The cultural atmosphere surrounding WSHA cannot be considered strong based on the investigation. Although the psychological comfort score was higher, the frequency of reminders was lower. This suggests that fostering a culture of surgical hand disinfection requires encouraging and promoting mutual supervision. Continuing hand hygiene initiatives through events such as World Hand Hygiene Day and Global Handwashing Day is also an effective way to strengthen this cultural atmosphere.

Through the implementation of several intervention measures, the WSHA score after the intervention was higher than before, but it still fell short of the mid-term assessment score. Behavior change requires ongoing training and intervention over an extended period. Automated hand hygiene reminder systems have been used to modify hand hygiene behavior (30), and with the advent of the AI information age, automated WSHA systems may also be used in the future. However, continuous supervision and training must not be neglected. Managers, in particular, should take a leadership role in guiding and supporting staff members.

## Conclusion

This study showed that clinicians showed less accuracy in implementing WSHA guidelines before the intervention. By raising awareness of hand disinfection and promoting a culture of hand hygiene, organized interventions were implemented to encourage the correct application of WSHA. Different groups were exposed to tailored interventions. In the future, it will be necessary to closely monitor and supervise WSHA practices to assess the long-term effectiveness of the current intervention strategies.

### Limitation of the study

There were several limitations in this study: (1) Demographic characteristics were not investigated in the pre-intervention and post-intervention stages due to the use of a double-blind method. (2)The study focused on the correct implementation of the WSHA procedure based on established standards, and only the outcomes of following the procedure were studied. The effects of WSHA when the procedure was not followed were not explored.

## Data Availability

The original contributions presented in the study are included in the article/supplementary material, further inquiries can be directed to the corresponding author.
